# M. Ribes on Section of the Flexor Tendons of the Knee in White Swelling

**Published:** 1845-01

**Authors:** 


*Section of the Flexor Tendons of the Knee in White Swelling.*—One of the most successful applications of tenotomy is unquestionably that of dividing the flexor tendons in certain cases of diseased joints, with the view of insuring the perfect quietude of the articular surfaces. It has been tried by several surgeons in white-swelling of the knee, and, we rejoice to say, with very promising success.

M. Ribes, a man of great experience, has recorded his opinion of the practice in the following words:

"The medical art is rich in therapeutic remedies for the relief of white-swelling of the knee-joint; but, in almost all cases, a simple cause has made them to prove utterly inefficient. This cause is the forced and permanent contraction of the flexor muscles of the leg. *Eh bien!* Why should we not perform, at the proper time, the subcutaneous section of the tendons of the Semi-membranosus, Semitendinosus and Biceps muscles, which keep up this uneasy state of things? By this easy operation, we may rationally hope not only to relieve the existing pain and distress, but also very materially to promote the formation of anchylosis, and consequently the cure of the disease. This simple and safe operation is already admitted and recognised by surgeons."

M. Ribes is one of those men whose name alone is worth a multitude of authorities, and whose simple approval will often weigh more than the most elaborate commendation of others.—*Ibid.*

